# Supplementary figures and images for: Ecological niche modeling for surveillance of foot-and-mouth disease in South Asia
Source: PLoS One. 2025 Apr 22;20(4):e0320921. doi: 10.1371/journal.pone.0320921 (PMC12013921; doi:10.1371/journal.pone.0320921)

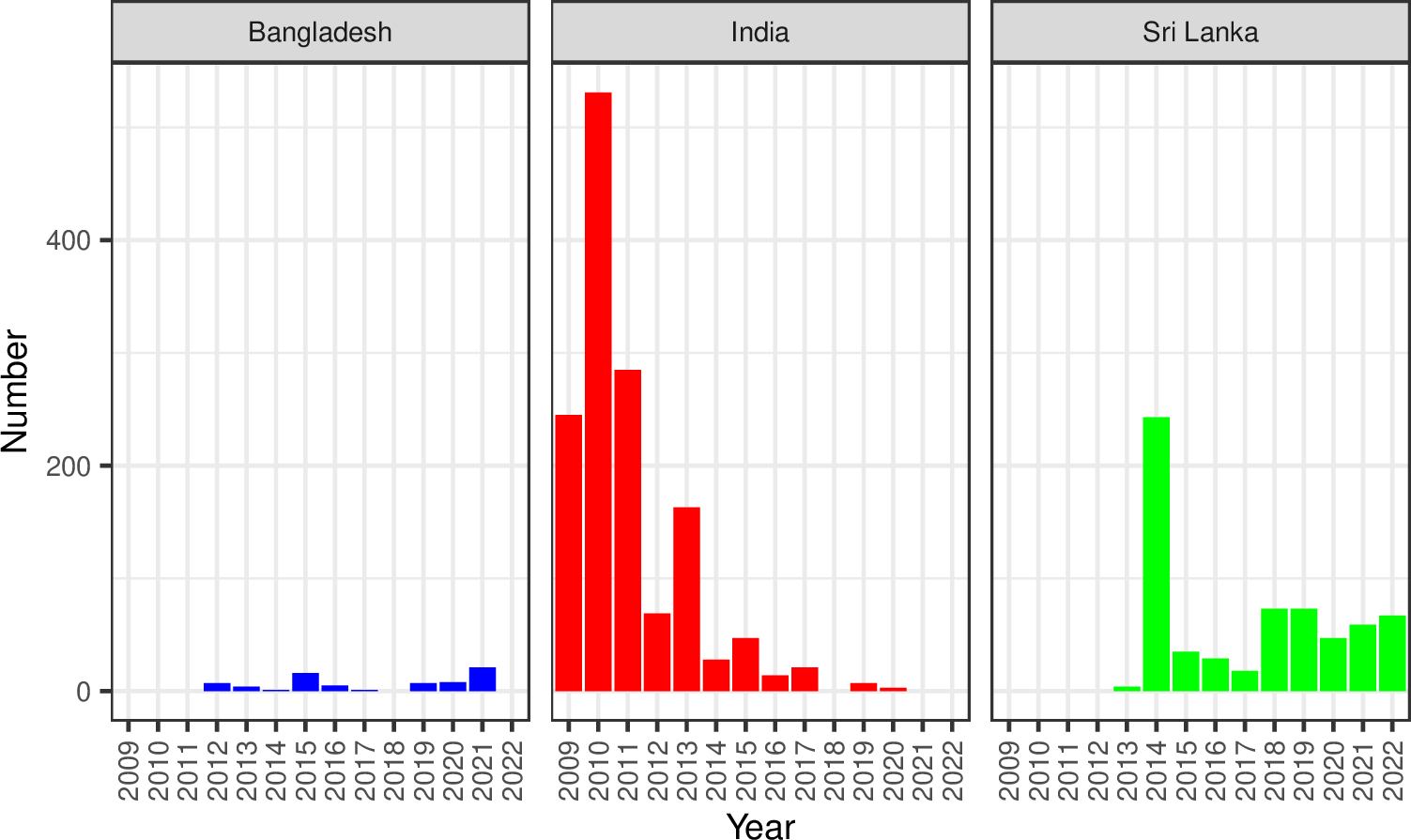

Supplement: S1 Fig — (TIF) [file pone.0320921.s001.tif]
