# Supplementary material for: Ecological niche modeling for surveillance of foot-and-mouth disease in South Asia
Source: PLoS One. 2025 Apr 22;20(4):e0320921. doi: 10.1371/journal.pone.0320921 (PMC12013921; doi:10.1371/journal.pone.0320921)
Supplement: S1 Table — (DOCX) [file pone.0320921.s002.docx]

# **Supporting information**

**S1 Table: Source of the different predictors, spatial resolution, and the data collected period considered in this study.**

| **Predictor** | **Source** | **Features** | **Reference** | **Spatial resolution** | **Period** |
| --- | --- | --- | --- | --- | --- |
| Historical climate data 19 bioclimatic features | WorldClim version 2.1 | Annual mean temperature,  Mean diurnal range,  Isothermality, Temperature seasonality, Max temperature of warmest month, Min temperature of coldest month, Temperature annual range, Mean temperature of warmest quarter,  Mean temperature of coldest quarter, Annual precipitation,  Precipitation of wettest month, Precipitation of driest month, Annual Precipitation,  Precipitation of wettest month, Precipitation of driest month, Precipitation seasonality, Precipitation of wettest quarter, Precipitation of driest quarter, Precipitation of warmest quarter, Precipitation of coldest quarter | [https://www.worldclim.org/data/worldclim21.html#](https://www.worldclim.org/data/worldclim21.html) | 5 minutes of Arc | 1970-2000 |
| Livestock densities | FAO, Gridded Livestock of the World database (GLW v4) | Cattle, buffalo, goats, sheep, and pigs livestock densities | <https://www.fao.org/livestock-systems/global-distributions/en/>. | 5 minutes of Arc | 2000-2015 |
| Normalized difference vegetation index | Copernicus land monitoring service |  | <https://land.copernicus.eu/en/products/vegetation/normalized-difference-vegetation-index-300m-v1.0> | 300m | 2014-2020 |
| Cropland | FAO Crop Land - Global Land Cover Share |  | <https://www.fao.org/land-water/land/land-governance/land-resources-planning-toolbox/category/details/en/c/1036355/> | 30 seconds of Arc | 2014 |
| Livestock production systems | FAO Global Livestock Production System |  | <https://www.fao.org/4/i2414e/i2414e00.htm> | 30 seconds of Arc | 2000- 2007 |
| Road network | Global Roads Inventory Project (GRIP) |  | <https://www.globio.info/download-grip-dataset> | 5 minutes of Arc | 2000 to 2015 |
