# Supplementary material for: Ecological niche modeling for surveillance of foot-and-mouth disease in South Asia
Source: PLoS One. 2025 Apr 22;20(4):e0320921. doi: 10.1371/journal.pone.0320921 (PMC12013921; doi:10.1371/journal.pone.0320921)
Supplement: Attachment — Submitted filename: Comments-Final to be submitted.docx [file pone.0320921.s004.docx]

**Preliminary observation:**

The manuscript entitled “**Ecological niche modeling for surveillance of foot-and-mouth disease in South Asia”** provides an interesting insight on risk factors associated for FMD in south Asian countries in general and India, Sri Lanka and Bangladesh in particular based on the data available. Of these countries India is in stage 4 of Progressive Control Pathway (PCP) of FMD, whereas other two countries are in PCP 1. Therefore the FMD control in India is at a very dynamic stage. The approach included use of appropriate models to normalize the possibility of outbreaks in different parts of these countries, as under-reporting of the outbreaks is common in the area due to different reasons. While the authors acknowledge the challenge of underreporting outbreaks, they fail to provide a transparent account of how the model compensates for this issue. Clarifying the methodology used to address underreporting is essential for enhancing the study’s credibility. This also included a variety of machine learning algorithms to identify the best fit using different (13) parameters responsible for disease/outbreak and risks associated.

**General observations**

# The English language used in the manuscript is good and easy to understand.

**Comments**

**Minor:**

The abbreviation of Ecological niche models (ENM) may be checked as they are written as EMN (lines 64 & 245). A concise explanation of how ENMs function and their relevance to FMD would significantly benefit the reader’s understanding. The figure S1 is missing in the supporting documents.

**Major:**

- The methodology used for analysis is very appropriate in terms of models (ecological niche model and machine learning tools and different parameters required for risk assessment of FMD. The manuscript mentions the use of random forest, support vector, and gradient boosting algorithms. However, it does not provide information on the selection criteria or performance metrics used for comparison. Also the methodology for handling duplicate outbreak occurrences is inadequately defined, lacking clear criteria for identification and removal. This ambiguity poses a risk of distorting the training dataset used for machine learning models, which could compromise the reliability of the results. A well-defined rationale and systematic approach to duplicate management are crucial to ensure data integrity and enhance model performance.
- The Indian FMD control programme has entered in to a very dynamic stage changing in every round of vaccination every six month. As shown in the manuscript that India contributes to the major niche in the region and remains a major source of viruses in the region, with a changed scenario this may not be true. The factors such as outbreak trend cannot be predicted, until the recent vaccination efforts, and trend of recent outbreaks are used to fit the model from India. This may also affect the prediction in neighbouring countries like Sri Lanka. Interventions during intensive vaccinations are very dynamic and difficult to predict due to changing herd immunity patterns every six months in different parts of the country. The implications of this data dependency should be thoroughly discussed to avoid misleading conclusions.
- Also degree of under reporting varies in different countries taken in the study and also different parts of a country like India needs to be predicted and differentiated (although manuscript discussed this at a point). Higher risk of predictions for India (0.75 as against 0.04 and 0.55 for Sri Lanka and Bangladesh respectively) compared to Sri Lanka and Bangladesh needs to be normalized in view of ongoing efforts, which have reduced the number of outbreaks significantly during the past two years on account of 3-4 rounds of mass vaccinations using a potent FMD vaccine and other measures.
- India is having a higher capability for diagnosis, virus identification and characterization, which sometimes gets reflected in scenario that most of the viruses originate from India (line 288-289) needs to be discussed in the manuscript.
- The analysis primarily considers environmental variables to predict FMD risk but may overlook other crucial epidemiological and socio-economic factors such as herd immunity levels, animal movement patterns, and vaccination compliance. These aspects are likely to be significant drivers of FMD outbreaks and could have strengthened the model’s predictive ability. Adding these non-environmental predictors could yield more comprehensive insights and potentially improve model accuracy. The absence of time-based analysis also limits the model's ability to predict outbreaks dynamically, making it less actionable for real-time decision-making.
