# Supplementary material for: Ecological niche modeling for surveillance of foot-and-mouth disease in South Asia
Source: PLoS One. 2025 Apr 22;20(4):e0320921. doi: 10.1371/journal.pone.0320921 (PMC12013921; doi:10.1371/journal.pone.0320921)
Supplement: Attachment — Submitted filename: Reviewer Comments for PONE-D-24-43580.pdf [file pone.0320921.s005.pdf]

Reviewer Comments for Manuscript Review

Manuscript Title : Ecological Niche Modeling for Surveillance of Foot-and-Mouth Disease in South Asia

This paper discusses how ecological niche modelling, coupled with machine learning, can be used to predict the risk of Foot and Mouth Disease (FMD) in the South Asian countries of India, Bangladesh, and Sri Lanka. The paper's main contribution is the development of a spatial risk map for FMD, highlighting areas of high risk in these countries. This information can then be used to inform and improve surveillance and control efforts for the disease in the region. I recommend that this paper be accepted **after major revision.**

Major Comments

1. Lack of Transparency on Data Comparability: A significant concern due to the lack of detail regarding data comparability between the three countries. While the authors mention their data sources, they do not adequately address potential variations in data collection, reporting standards, and diagnostic confirmation practices. This lack of transparency could misinterpret the risk predictions and undermine the study's overall reliability. The authors need to provide a more detailed account of the data used for each country, acknowledging potential limitations in comparability. This might involve describing the data collection methodologies used in each country, discussing any inconsistencies in data reporting formats or definitions, and addressing the potential impact of variations in diagnostic testing practices.

2. Potential Data Leakage: Can the authors please check if there was no potential data leakage leading to overly optimistic model results? Performing feature selection before splitting the data into training and testing sets can lead to data leakage, especially if the feature selection process includes looking at the entire dataset, which allows information from the test set to influence feature selection. The Boruta feature selection method was applied to the entire dataset before the 80/20 split and cross-validation. Since Boruta uses a random forest classifier with access to all data, it implicitly gains information from the test set, thus creating a risk of data leakage. This could lead to overly optimistic performance estimates when training and validating the model.

3. Class Distribution in Model Evaluation: The authors evaluated the model using various evaluation metrics commonly used for imbalanced data. However, we did not see any mention of the class distribution, such as a ratio of 30% minority to 70% majority. How was this balance adjusted, and at what stage was the ratio modified? Oversampling before the data split will also introduce data leakage.

4. Caret Package Use: It is generally good practice to explain the use of the caret package for hyperparameter tuning in the manuscript. This explanation provides transparency and helps others understand your methodology, which is crucial for reproducibility and scientific rigour.

5. Clarification on Variables and Feature Selection:

- The authors did not indicate the total number of variables considered. Additionally, how many variables were removed after correlation filtering, and how many variables remained, potentially around 23?

- Please clarify that 15 predictive variables were selected from the Boruta algorithm out of 23 total variables. How many times was this process run? Would the results still be consistent if it was run multiple times, such as 50?

6. Model Run: How many times were the models run? Was it a single run or multiple runs? Please clarify and provide details on how the model was robustly evaluated.

7. Comparability of Predicted Risk Across Countries: Is the lower predicted risk in Sri Lanka and Bangladesh due to differences in data availability? If so, these countries should not be directly compared. The authors need to highlight this potential limitation to avoid misleading interpretations. To address this issue, the authors should:

- Explicitly discuss the availability and quality of data for each country.

- Acknowledge the potential impact of data variability on the comparability of predicted risk estimates.

- Suggest further research to enhance data collection and reporting in all three countries, particularly in Bangladesh, where data was sourced from various publications instead of a central source.

By transparently addressing these limitations, the authors can offer a more nuanced and reliable assessment of FMD risk in South Asia, leading to more informed and effective control strategies.

Minor Comments

- Many terms were abbreviated earlier in the document but were still mentioned in their full form later on. For example, the World Organization for Animal Health (WOAH) was abbreviated in the introduction but was referenced again in the methods section. Additionally, terms such as Random Forest (RF), Gradient Boosting (GB), and Support Vector (SV) machine were used multiple times throughout the text.

- Please verify whether the authors used Support Vector Machines (SVM) since it is uncommon to refer to them simply as "Support Vector (SV)."

- It is important to specify the Gradient Boosting (GB) type used. Is it XGBoost (XGB)?

-Specifying the R package version and putting proper citations is a good practice. Specifying the package version ensures that the analysis can be reproduced accurately, as different versions may have updates or changes that could affect results.

-Algorithm Selection: Please justify the algorithm selection. While the use of multiple machine learning algorithms is commendable, the manuscript would benefit from a more detailed justification for selecting these specific algorithms. Explaining why random forest, gradient boosting, and support vector machines were chosen over other potential methods would strengthen the methodological rationale.

- Readability: Regarding structure and readability, the sentence, "Using a multi-algorithm machine-learning ensemble that includes random forest, support vector, and gradient boosting, 15 predictive variables (i.e., livestock densities, land cover, and climate), 660 FMD outbreaks from 13 years (2009-2022) in the regions including the outbreaks from India, Bangladesh, and Sri Lanka, we identified that Sri Lanka and Bangladesh appeared to have low to medium outbreak risk in the range of 0.04 to 0.55. India was used to fit the model," can be improved. It could be clarified by splitting it into two sentences after "low to medium outbreak risk in the range of 0.04 to 0.55."

- Table 1 Explanation: The authors reported model performance; however, Table 1 lacks an explanation of what is being presented. Is it a standard deviation (SD) or a 95% confidence interval (CI95)? Furthermore, please check if 94.45±0.01 is derived from the SD result, like 0.9445±0.01. To clarify, 94.45±0.01% = 0.9445±0.0001 on a 0-1 scale, while 0.9445±0.01 on a 0-1 scale corresponds to 94.45±1% in percentage terms.

- Consistency in Metrics: Please ensure consistency in the results table. All metrics should be reported in the same format throughout the manuscript, as mixing percentages and 0-1 scales can lead to confusion.

- Implications for Control Strategies (Optional): Expanding on Implications for Control Strategies: While the discussion mentions the need for a regional approach to FMD control, it could benefit from a more detailed exploration of specific control strategies informed by the study's findings. The authors could discuss how the identified risk factors and high-risk areas could guide targeted interventions such as vaccination campaigns, movement restrictions, and surveillance efforts. Discussing practical applications of the research would enhance the discussion's impact.

<u>Assessing the Manuscript Against PLOS ONE Publication Criteria</u>

1. Originality: The study appears to present original research**.** The authors have applied ecological niche modelling and machine learning techniques to predict FMD risk in South Asia, which seems to be a novel application in this context.

2. Previous Publication: Verifying this independently through a comprehensive literature search and plagiarism check is essential.

3. Technical Standards and Description: The experiments, statistics, and analyses were conducted to a high technical standard. The authors have clearly outlined their methodology, including data sources, processing steps, model selection, and evaluation metrics.The description of the methods is generally detailed, enabling replication and scrutiny by other researchers. The authors have provided specific details about the software packages used, data aggregation and resampling procedures, and model training and validation techniques.However, as discussed previously, a key area requiring improvement is the lack of transparency regarding data comparability between the three countries. This aspect needs further elaboration to ensure the robustness and reliability of the findings.

4. Conclusions:

- The conclusions presented in the manuscript appear to be supported by the data and analysis. The authors have identified specific high-risk areas for FMD in each country and have highlighted the key risk factors associated with FMD occurrence.
- The discussion section reasonably interprets the findings, relating them to existing knowledge and highlighting the implications for FMD control in South Asia.
- However, the conclusions could be strengthened by providing more specific recommendations for control strategies based on the identified risk factors and high-risk areas.

5. Intelligibility and Language:

- The manuscript was written in clear, intelligible English and followed a logical structure.
- The language used is generally appropriate for a scientific publication, although some minor stylistic improvements might be suggested during the review process.

6. Ethical Standards:

- Generally, the data analysis did not need ethical decorations. In case of interviewing , the authors need to provide proper consent forms.

7. Data Availability:

- The manuscript explicitly states that all data underlying the findings are fully available without restriction. The authors have provided details on where the data can be accessed, including references to government websites and published studies.

Overall, the manuscript appears to have the potential to meet the criteria for publication in PLOS ONE, but requires revisions. Once these revisions are addressed, the study could contribute valuable insights to the field of FMD research and control in South Asia.
