# Supplementary material for: Ecological niche modeling for surveillance of foot-and-mouth disease in South Asia
Source: PLoS One. 2025 Apr 22;20(4):e0320921. doi: 10.1371/journal.pone.0320921 (PMC12013921; doi:10.1371/journal.pone.0320921)
Supplement: Attachment — Submitted filename: Reviewer Comments and Recommendation for Acceptance.pdf [file pone.0320921.s007.pdf]

## Reviewer Comments and Recommendation for Acceptance

We appreciate the authors' efforts in revising the manuscript **Ecological Niche Modeling for Surveillance of Foot-and-Mouth Disease in South Asia."(PONE-D-24-43580_R1)"** The authors have successfully addressed all major concerns, providing clear explanations, methodological improvements, and additional details that enhance the transparency and rigor of the study. Below is a summary of the key reviewer concerns and how the authors have responded effectively.

### 1. Transparency on Data Comparability

The initial concern was the lack of detailed information regarding how data were collected and compared across India, Bangladesh, and Sri Lanka. The authors have now included comprehensive explanations about data sources, reporting standards, and diagnostic confirmation practices, ensuring greater clarity and reducing potential misinterpretations.

### 2. Comparability of Predicted Risk Across Countries

The reviewer questioned whether disparities in data availability influenced differences in predicted risk levels rather than actual disease risk. The authors have acknowledged this limitation and further discussed how surveillance capabilities vary across the three countries. Additionally, they have suggested future research to improve data consistency, particularly in Bangladesh, where centralized surveillance is lacking.

### 3. Potential Data Leakage

Concerns about possible data leakage were raised, as feature selection was performed before splitting the data into training and testing sets. The authors have addressed this by explaining that cross-validation helps mitigate this risk, and they have now included this as a potential limitation to ensure transparency.

### 4. Class Distribution in Model Evaluation

The absence of an explicit mention of class distribution in model evaluation was noted. The authors have now clarified that a **1:1 case-control ratio** was used, ensuring balanced representation of presence and absence cases. Additionally, ROC curves confirmed that no class imbalance was present.

### 5. Use of the Caret Package for Hyperparameter Tuning

The reviewer suggested that the authors explicitly mention their use of the **caret** package for hyperparameter tuning. The authors have acknowledged this and have now provided a brief explanation for transparency and reproducibility.

### 6. Clarification on Variables and Feature Selection

The reviewer requested details on the total number of variables considered and the number removed after correlation filtering. The authors have now included a supplementary table listing all **24 variables**, clarified the **9 variables removed due to correlation (>0.9)**, and confirmed that **15 variables** were ultimately used in the analysis.

### 7. Model Runs and Robustness Evaluation

The reviewer inquired about the number of times the models were run and the robustness of the evaluation process. The authors have now specified that each model underwent **10 runs of 10-fold cross-validation**, ensuring reliability and reducing overfitting. This additional detail strengthens the credibility of the model performance results.

## Final Recommendation

With these revisions, the manuscript now provides **a more transparent, methodologically sound, and well-documented study** on ecological niche modeling for foot-and-mouth disease surveillance. The authors have successfully addressed all concerns with thorough explanations, added details, and clear acknowledgments of limitations.

Given the improvements, I **recommend accepting this manuscript** for publication. This study offers valuable insights into regional FMD risk assessment using machine learning, and the refinements made in response to the reviews significantly enhance its impact and reliability.

Best wishes,

Reviewer 2
